# So, You Think You Have an Idea: A Practical Risk Reduction-Conceptual Model for Academic Translational Research

**DOI:** 10.3390/bioengineering4020029

**Published:** 2017-04-04

**Authors:** John Schwartz, Christopher Macomber

**Affiliations:** 1 AcuityBio, Inc., 200 Upland Road, Newton, MA 02460, USA; 2 Surgical Specialists of Minnesota, Abbott Northwestern Hospital, Minneapolis, MN 55404, USA; chris@drmacomber.com

**Keywords:** translational bioengineering, translational research, medical technology, innovation, health care, risk, conceptual health model

## Abstract

Translational research for new drugs, medical devices, and diagnostics encompasses aspects of both basic science and clinical research, requiring multidisciplinary skills and resources that are not all readily available in either a basic laboratory or clinical setting alone. We propose that, to be successful, “translational” research ought to be understood as a defined process from basic science through manufacturing, regulatory, clinical testing all the way to market. The authors outline a process which has worked well for them to identify and commercialize academic innovation. The academic environment places a high value on novelty and less value on whether, among other things, data are reproducible, scalable, reimbursable, or have commercial freedom to operate. In other words, when investors, strategic companies, or other later stage stakeholders evaluate academic efforts at translational research the relative lack of attention to clinical, regulatory, reimbursement, and manufacturing and intellectual property freedom to operate almost universally results in more questions and doubts about the potential of the proposed product, thereby inhibiting further interest. This contrasts with industry-based R&D, which often emphasizes manufacturing, regulatory and commercial factors. Academics do not so much choose to ignore those necessary and standard elements of translation development, but rather, they are not built into the culture or incentive structure of the university environment. Acknowledging and addressing this mismatch of approach and lack of common language in a systematic way facilitates a more effective “translation” handoffs of academic project concepts into meaningful clinical solutions help translational researchers more efficiently develop and progress new and better medical products which address validated needs. The authors provide an overview and framework for academic researchers to use which will help them define the elements of a market-driven translational program (1) problem identification and validation; (2) defining the conceptual model of disease; and (3) risk evaluation and mitigation strategies.

## 1. Introduction

Cohrs, et al. described translational research in medicine very well when they said: “TM [Translational medicine] as an interdisciplinary branch of the biomedical field supported by three main pillars: bench, bedside and community. The goal of TM is to combine disciplines, resources, expertise, and techniques within these pillars to promote enhancements in prevention, diagnosis, and therapies.” [1].

The target audience for this work are scientists, clinicians, and technologists hoping to translate technologies they develop at their bench to be used at a patient’s bedside will benefit by better understanding how these technologies and therapies are currently commercialized and what forces have led to the existing paradigm.

Currently, the most likely commercialization scenario for translational products is through acquisition and marketing by one of the few large medical device or pharmaceutical companies. Billion dollar total revenues of the dominant, entrenched public companies. A solution to be deemed valuable it must be seen tofit into the established business models of these large companies in order to attract strategic investment, co-development or result in acquisition. Market value, project development, and commercialization risks are the lens through which potential partners and acquirers view all potential translational projects. Shopping for new pipeline assets by large medical device or pharmaceutical companies can be good news for translational teams in academia if they can mitigate perceived risks and can communicate clearly what the value proposition of their project is to the large company and the broader market. The main issue is how to bring the core areas of expertise and a framework to provide them into the academic model without disrupting the creative processes that lead to these successful innovation.

The life science industry has for many years been working on traditional business models based on R and D and innovation where physicians have been the target customer and third party payers willing to reimburse the cost for products with incremental improvements. The efficacy and safety of the products had been the most important criteria for their commercial success, not innovation or cost-effectiveness. However, over the last ten or so years, there has been a marked shift to “value-based healthcare,” which has led to companies revamping their business models and has resulted in industry-wide mergers and consolidation given a lack of “value”-driven innovations.

The market pressures on all medical device and pharmaceutical firms are pressing them to deal with how to improve their financial bottom line. These factors are forcing companies to reevaluate how they compete in the marketplace. Many of these large companies seek to shed fixed costs for R and D and to acquire products and services for their franchises in order to package and market them to their established call points. The result of the changes in the industry business model is that the market is essentially now controlled by a few public companies. For example, in the last two decades, 60 pharmaceutical companies have become just 10 Big Pharma companies. This consolidation has helped Big Pharma gain more muscle to influence regulation while simultaneously diminishing the competition [2]. Increased regulation is squeezing the growth of medical device company revenue [3]. In 2013, the Medical Device Excise Tax, a provision of the Affordable Care Act (ACA) that assesses a 2.3% tax on medical devices, took effect and the impact has been significant [4]. Medical device companies are subject to increased pricing pressures as large healthcare entities and Group Purchasing Organizations (GPO’s) leverage their enhanced bargaining power.

These factors have increased the cost of doing business and serve to decrease margins in an already highly-competitive medical device industry. Some critics argue that an over-reliance on M and A in the pharmaceutical industry is ultimately unsustainable because of the damaging effect it has on the industry’s R and D capacity. M and A may streamline the new combined organization, making it “more efficient” and wring out redundancies. The result is often that R and D activities are curtailed and projects with long timelines are eliminated secondary to a nearer term and limited internal pipeline capacity and the need for new revenue generation. These large companies now look to acquire translational assets either from academia or via the acquisition of smaller innovative companies with existing or groundbreaking products in a few franchise areas in order to white label and scale up manufacturing of the technology.

As a result, consolidation and the requirement by public companies to deliver strong share price performance has resulted (with some exceptions) in a dearth of acquisitions and funding for innovative translational research and start-ups offering innovative products. There has been a shift to deploy corporate capital in the pursuit of lower P and L risk technology acquisitions at later stages of development and resistance of these market leaders to acquire new, expensive, clinically, unproven cost effectiveness, or other form of “value” at an early stage, or non-revenue producing technology. This is a market opportunity for academia. By incorporating methodology typically employed in early-stage companies looking for strategic partnership or acquisition, academia can produce the equivalent of “later-stage” technology that is more appealing to, and more in line with, the technology desirable by the acquirer.

To take advantage of this opportunity, academics need to recognize what drives licensing and acquisitions at these larger companies. As these companies tend to be more risk averse to things that they know will incur significant costs and time to remediate if found deficient, the translational scientist, or a supporting team/framework at their institution, needs to address these concerns up front in order to attract interest. From the large company perspective, quality-and-risk assessment, physical plants, personnel, processes, procedures supplier networks are extremely important. This is because these areas are a significant focus for the FDA and other regulatory bodies. Companies can incur millions of dollars in expenses to remediate issues with acquired assets even before they can offer those products for sale.The reimbursement strategy must be addressed up front to some extent even though they are historically not in the academic setting. Nonetheless, early assessment of reimbursement risks and rewards help in identifying and presenting the best candidates for corporate collaborators and acquirers.

To engage in successful relationships with these large companies, a translational scientist/entrepreneur and/or their supporting academic innovation team need to ensure that the future product will provide a demonstrable positive outcome and sufficient return on investment in order to garner the right attention.

Academic hypothesis-driven development of new knowledge is vitally important and still represents the main driver of new innovative ideas the life sciences and healthcare market relies upon. However, academic researchers are primarily engaged in the development of new knowledge, and have different skill sets, resources, and incentives compared to those involved in later stage medical technology development.

Academic translational efforts typically rely on grant funding and have success determined by innovation, not commercialization, the number of high-impact publications, successful education of future researchers, and academic recognitions. This has been the backbone for the development of many new and innovative medical products. The challenge to effective translational efforts at universities and medical centers has frequently been that academic researchers often understand that the market opportunity or critical unmet medical needs for new technology in their research space could be very large. However, they may not have the resources, training, or incentives to develop data supporting necessary critical engineering/manufacturing, clinical evidence, or regulatory requirements to help position their innovations to become finished goods ready for human testing or a final product ready for regulatory approval and sale. The challenge is to determine what stage a researcher’s institution (e.g., proof of concept, large animal experiments, clinical trial, etc.) would like to push their innovation to and how best to position it for that acquisition. That is a decision best determined by the researcher, their team in collaboration with their institution, co-developers, sponsors, and collaborators. The academic institution should not focus on becoming a manufacturer or start-up, but they should appreciate the key steps and risk factor variables that determine a successful product from one that fails.

Therefore, the proper evaluation of any new medical technology needs to follow a market-based framework that insures alignment of “translational” innovation with validated de-risking steps addressing the wants and needs of likely corporate licensee, partners, co-developers, or acquirers.

## 2. Understanding the Term “Translational Research”

It is important to keep in mind that translational innovations do not need to result in merely drugs or devices and encompasses several transitions towards commercialization, and beyond. While we will focus on the earlier stages of translational activities, it is useful to see the whole myriad of “translational” activities in order to see where and how your project is seen to fit into the standard workflows for development and commercialization. This will better enable you (as scientist, clinician or technologist) to focus your development resources when working to de-risk a project, as we detail more clearly below, and during the process of promoting greater clarity for a project’s value proposition to external stakeholders.

**Figure 1:**
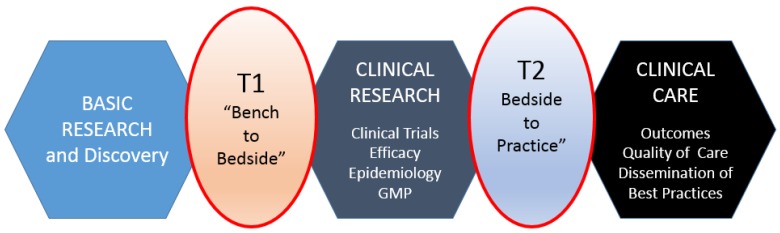
Schematic transitions from the “Bench to Bedside” (**T1**) and “Bedside to Practice” (**T2**) translational medical research and development model. The process of invention and then development is bidirectionally promoted by academic inquiry, as well as astute clinical observations. “Bench to Bedside” is catalyzed by first principles inquiry and the “Bedside to Bench” direction is catalyzed by a clinical observation and an articulated “if only I had….” to solve this clinical problem or fill this unmet need in my practice of medicine.

**Figure 2:**
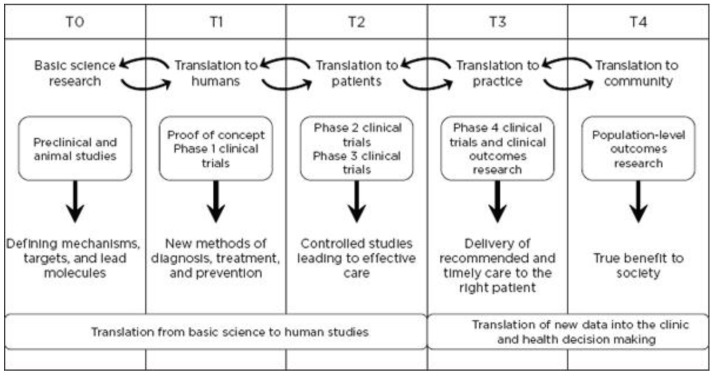
Operational phases of translational research in greater detail (**T0**–**T4**), Reprinted by permission from Macmillan Publishers Ltd.: Nature Medicine [3] Copyright 2012 [3].

Translational research, for the purposes of this article, includes two areas: (1) bench to clinical testing (“bench to bedside”) and (2) clinical practice to scaling best practices of clinical care. Bench to clinical testing is shown as T1 in Figure 1 and is the process of taking basic discoveries generated during research, and in preclinical studies, and their reduction to practice through the development of trials and studies in humans. The second area is T2 in Figure 1, concerns research aimed at enhancing the adoption of best practices in the community as well as the development and deployment of cost-effectiveness treatment or prevention strategies (“bedside to practice”). Translational research can include a vast array of new processes, treatment strategies, information technology applications, and quality improvement projects, to name a few. The element that ties these together at a higher level is the understanding of the market need, or problem area focus. Successful translational research needs to take into consideration the business, regulatory, reimbursement, and adoption elements needed to help an innovation become a scaled commercial success as illustrated in more granularity in Figure 2. This is not to say the academic institution needs to know how to accomplish the steps needed in the later stage aspects of development and manufacturing, however, it is important to understand that there are critical steps, functions or activities that, if accomplished early in the research process and/or accomplished to the right standard or requirement, can have a dramatic improvement in success during later stage processes. This is why it is important to have the proper team and collection of experts available to support the research.

## 3. You Have an Idea for Academic Translational Research; Now What?

The following sections are intended to provide a basic framework for how you can begin to evaluate, validate, and develop plans to de-risk your potential translational project and medical product. The three concepts we propose that are critical in designing, validating, executing, and communicating your translational project are:

1. Concise and validated problem definition;
2. The conceptual health or conceptual innovation model; and
3. Risk reduction in preparation for a start-up or licensing opportunity.

The following sections introduce how we propose bridging the academic researcher trying to develop projects from the bench to the clinic, i.e., T1 transition. The foundation of our process is based on stepwise risk reduction and conceptual models of disease, which will help simplify design, execution, and communication of the ways in which you have considered and addressed project risks and refine the value proposition of your project. This will aid you in communicating with, and enticing, large companies to commercialize the product or service your multidisciplinary team plans to develop. At each step of the process, there are a series of questions that a team/researcher should ask that assist in drilling down on core elements of risk. It begins with the initial concept evaluation and definition of the problem, followed by framing the problem and solution in the context of a conceptual health model—which serves to fit the solution into the larger market so as to begin to work on the third and final process of risk reduction.

### 3.1. Initial Translational Product Concept Evaluation

Translational teams must efficiently validate and then pursue a focused and a well-defined development path to develop commercially viable and clinically meaningful product or service. The start of your translational campaign needs to begin with a clear articulation of a validated problem/market opportunity. The easiest way to start this process is to begin with a simple question: “Does my innovation solve/address a valuable unmet need or problem?”

This question helps to focus the goal of the innovation and help separate broad research from those with a targeted potential pathway to commercialization. We have seen a fair number of academic research projects fail at commercialization as the innovation had multiple potential opportunities that may have been possible. However, in pursuing a commercialization opportunity, the team was unable to define a focused problem area and how their innovation was the solution. As a result, those evaluating the innovation for license or acquisition are unable to make the connection between the disorganized collection of data, results, patent claims, etc., and how that would translate into their focused, narrow, internal commercialization/marketing channels. In addition, by focusing on a single problem area and how your innovation is the solution, you may be in a better position to identify research pathways, experiments, or validation steps to bolster the case for that one indication. This is not always the case; however, in certain situations, identifying simple or lower cost experiments, design issues, etc., at an early stage build a better case for your innovation’s capabilities and result in cost savings at later stages of development. This is critical as it enhances the business case, may improve the design or quality of your innovation and possibly uncover new patentable claims that improve the intellectual property position of the innovation.

A takeaway is that although your research may have a broad list of applications, including the potential to be a platform technology, it is critical to focus on a narrow problem to solve in order to create a business case and presentation that allows an acquirer to see the value. They will ultimately see the broad applications, which is part of their due diligence process, but the academic team must prioritize and show a narrowly-focused solution to a specific problem with an understandable and viable business case. This will ultimately result in more excitement and interest in the innovation and, naturally, will flow to the potential alternative applications once the interest is there. It is a common misstep to assume showing multiple applications increases interest in a concept. Success in the market is based on a narrow indication, given the requirements associated with achieving regulatory approval and reimbursement.

It is critical to understand what you at your specific institution can accomplish with existing strengths and limitations of your resources, personnel, and environment. You should try to define the knowledge gaps and the need to bring in additional resources outside your institution to complement these gaps in your risk reduction plan, i.e., clinical practice key opinion leaders, patient advocacy groups, reimbursement consultants, engineers, manufacturing and clinical contract research organizations, industry experts, regulatory consultants, etc. Questions that help identify the gaps include: “Can you assemble a multidisciplinary team?” “Do you have the resources and technical skills to address development risks?” “Who will direct the project and who will communicate and negotiate on behalf of the project?” Having someone on-staff who acts as a project manager of the multidisciplinary team really helps drive rapid and efficientproject progress.

Defining exactly what you will and will not address with your treatment, device, or intervention is important in defining the scope of the project, as well as the resources and time required to achieve success. When these concepts are applied to even the earliest of research projects, it ensures the research team are focused on a validated process to increase their likelihood of reaching the end goal of commercialization and aligning the research the goals and needs of the eventual acquirer of the solution

### 3.2. Conceptual Model for Innovation

The second element of this process after addressing the standard risks, shown in the project risk grid in Figure 4, is to understand as the conceptual model of disease; which better defines the addressable market opportunity for your innovation or technology. While this is a common technique in mapping disease processes, we have found it very valuable to pursue the same exercise to map a disease, problem-area, workflow, or process that a given innovation or drug is intended to address. Although a researcher or team may not have answers to all of these risk areas at the beginning of the project, we have found that incorporating that mindset early in the process helps to prevent many common missteps or project directions that would alter elements of the project that might otherwise result in a successful license or start-up later on. While in our own practice we pursue a much more detailed analysis, for the purposes of this article, and to introduce the broad concepts, we are providing a higher-level overview.

One of the best methods we have found to begin going through a series of questions. Depending on the nature of your project, you will look to map out the process, disease lifecycle/model, or workflow of the issue that your innovation is looking to address. Key questions at this stage include: “What are other options in the market to solve the problem?”, “Does your innovation have the potential to disrupt a presently available solution in the market?”, “If so, are they a threat or opportunity for licensing?”, “What were the regulatory requirements for competing or similar technology that solve the same problem?”, “What is the size of the market for the problem your addressing?”, “What realistic percent of the market would your innovation be able to serve?”, and “What is the cost of existing options in the market and does your innovation provide a cost savings?” 

The conceptual model of disease is a well described concept and helps to define the risks and rewards for your technology when applied to the current state of “standard of care.” Chronic kidney disease is a useful example. The conceptual model of chronic kidney disease (CKD) was developed by the National Kidney Foundation's Kidney Disease Quality Outcome Initiative (NKF-KDOQI) [5,6]. This model includes concepts of definition, staging, outcomes, and treatment, as well as risk factors for the development, progression, and complications of CKD. Treatments are available for patients with risk factors and for each stage of CKD; these include slowing the progression of kidney disease, preventing, and treating the complications of decreased glomerular filtration rate, and reducing cardiovascular disease risk factors and treating cardiovascular disease. In principle, measures to improve the prevention, detection, and treatment could reduce adverse outcomes, improve the quality of life, and prolong the survival of individuals with CKD.

**Figure 3:**
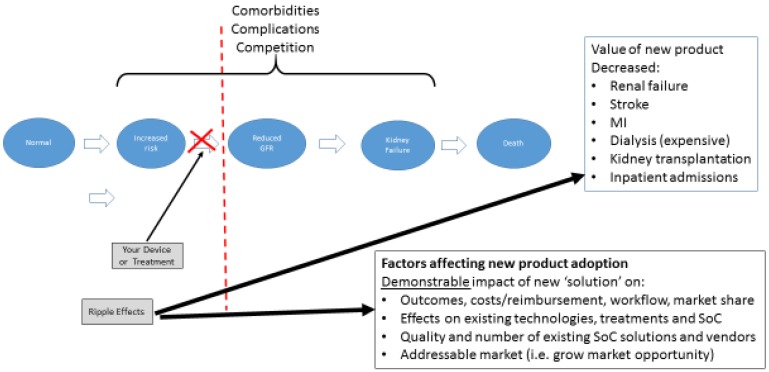
Natural history of disease and nodes for intervention and innovation.

The example cited here is one of the best represented conceptual health models—end stage renal disease (“ESRD”) that follows from CKD. This disease process is well understood and begins with an otherwise normal patient (foregoing consideration of other underlying medical problems or comorbidities to keep the example simple, of course). As the disease progresses through several well-defined steps, one can map the inputs and outputs at each step related to addressing the result of the forward movement. In Figure 3, you can see the transition from “Increased Risk” to “Decreased Glomerular Filtration Rate” (GFR) as a major step in the process of disease. In this case, it represents the first time true pathologic alteration occurs, indicating the disease process is now present.

In evaluating your technology or innovation using this methodology, you would determine where in this process it would fit in. This, then, allows you to conduct a simple market and competitive analysis. In addition, provides basic information regarding:

- How your technology impacts/disrupts the process downstream including:
  ○ Impact on the disease, process, or workflow outcomes (i.e., decreased renal failure, reduced need for dialysis);
  ○ Impact on technology, competitors, etc., whose market exists at those later stages;
  ○ Potential strategic partners or acquirers; and
  ○ Outcomes your innovation would have on clinical, financial, or other performance indicators.
- What steps would be required to show your capabilities at that given step by looking at what others have done or needed to prove (i.e., clinical evidence needed, regulatory or reimbursement requirements, etc.)

In general, if an innovative concept achieves an equivalent level of efficacy, improvement, etc., and does not reduce cost is much less likely to be adopted. Increasingly, a key element of this process is not just a demonstration of efficacy, but also the ability of something new to be more cost effective. By using this process, it allows one to explore not only if the new technology can reach the same level of efficacy as existing options, but also if it does it at a reduced cost.

Anexample of this issue, would be a hypothetical technology that had the ability to detect decreasing GFR (or early impaired renal function) at an earlier stage than any existing technology/diagnostic on the market, but was substantially more expensive. However, let us assume for this example that the new technology uncovered the alteration in GFR at such an early stage that a patient would be identified and treated and not progress to ESRD with a need for costly dialysis or kidney transplant, or that their progress to that stage could be delayed by some significant amount of time. In that case, the upstream cost of the diagnostic would be potentially warranted as it decreases the number of patients needing dialysis or transplantation, a tremendous cost to the health care system. Due to this analysis, you would be better able to then understand what kind of evidence would be needed to support those potential outcomes and better position your translational work going forward. Once you develop this conceptual model and begin to identify the project risks, you have made significant progress towards developing a thoughtful development program for your academic translational project and directly address the checklist corporate and venture investors use to measure the viability of your proposed project.

At this point, the project has already overcome several of the major hurdles that cause academic translational projects to fail. It is important to note that while you have made significant progress, there are still additional commercial criteria and constraints to address in detail as part of your project planning including: patentability, “freedom to operate”, validation of accessible market size, competition, technical feasibility, regulatory approval pathway, clinical testing feasibility and costs, reimbursement strategy and customer needs validation. The information developed by addressing these questions will allow you to develop an objective rationale to proceed with development and structures your project planning in a way that reduces risks and addresses questions that industry partners, investors, and granting agencies want to know. Each of these points is a significant project in itself and a practical “how to” is outside the scope of this article. Our point in highlighting these matters is to sensitize the academic team to the fact that there are important matters that they will need to address which are outside their own areas of expertise, requiring partnering with or hiring resources who have the professional competency to address these matters, i.e., patent and corporate lawyers, reimbursement experts, technology licensing, etc. This is what we mean by “interdisciplinary team”.

### 3.3. Understanding Risk

**Figure 4:**
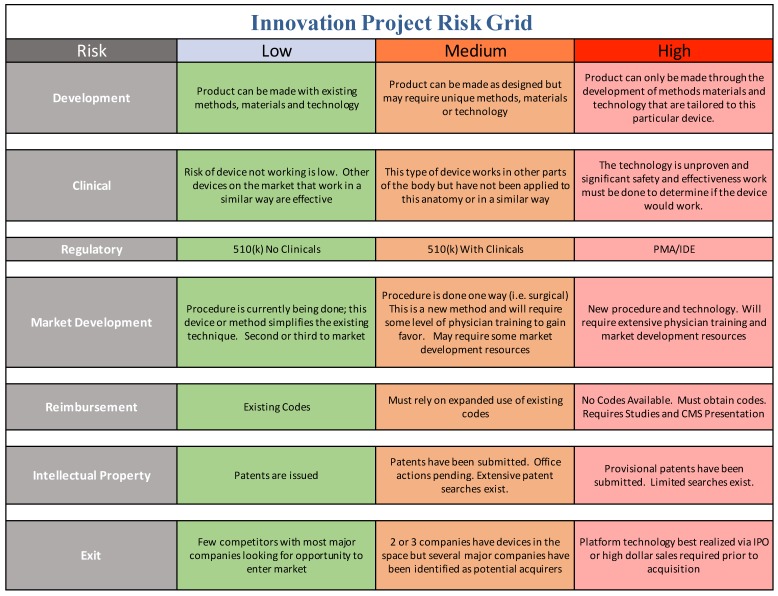
Medical device innovation project risk grid. This outlines the risks of any medical device project from prospective investor and co-development partner. Drug and diagnostic risk grids overlap the medical device risk grid categories but may include additional categories beyond the scope of discussion of this article.

Market opportunity is one parameter that industry and innovators focuses on, but risk is the other parameter which can be less clear to the academic translational scientist. In order to communicate with industry, you need to speak the language of industry, addressing both market opportunity and risk mitigation. We offer a practical tool for the translational team to use in order to help address risk in a manner that industry can accept (Figure 4).

Risk is a well-defined term and risk mitigation is a key part of communicating to industry. For example, risk mitigation in the medical device industry has published standards listed in ISO 14971 “Risk Management for Medical Devices” [7]. Risk per ISO 14971 is defined as the combination of the probability of occurrence of harm and the severity of that harm. The intent behind “Risk Management” is to identify, evaluate, analyze, assess, and mitigate potential product issues. We, again, believe a series of question-based assessments is helpful. Common questions at this stage include: “Does the innovative concept work? And how do you know?”, “Does it represent a significant improvement over existing solutions? How much of an improvement?”, “If it is not a significant improvement, is it intended to be a cheaper alternative?”, “If you lack confirmatory evidence, how much evidence do you think is needed to prove the solution and what is required to collect it?”, “How would the solution make money, how long until it made money, and who does it make money for?”, “Are their novel aspects to this solution that are patentable?”, “Does it have multiple applications? Which one is the biggest and or nearer-term opportunity (e.g., comparing overall market opportunity with the value of a smaller label but faster speed to market or partnering opportunity)?”, and “Would this solution as designed have any regulatory requirements? Animal studies, human clinical trial, etc.?”

Potential acquirers and regulatory bodies are highly risk averse. Successful translational projects need to focus on a risk mitigation plan. The academic team’s focus on the recognition of risk and having a plan of action for its step wise reduction aligns the academic research team’s perspectives closely with the industry and investors. Primarily, a risk reduction plan helps promote positive interactions during industry project portfolio reviews when selecting projects for sponsorship or acquisition. This will also yield a side benefit helping garner favorable grant review, or when considering a start-up company/spin-out from your institution.

The academic innovation team should be continually evaluating the project plan and goals through the lens of structured risk mitigation defined in terms that industry recognizes. This can be a daunting challenge for academic teams, depending on the level of resources, industry experience, entrepreneurial and business expertise available. We have developed a more academic user-friendly tool compared to the ISO standard and use it on every project or start-up we are involved in, as shown in Figure 4.

All projects have their unique aspects but we believe that this conceptual approach ought to be a center point of translational research. The risk grid tool below is introduced here as a conceptual model of how to systematically identify, and mitigate your translational project risks. Your team should use this approach to fill in the matrix using your best efforts and consult with expert resources where you cannot. Always keep in mind that you do not have to have answers to all elements of risk and questions that stem from them. You just need to demonstrate that you have thought about it and have a plan to address it, even if you believe that risk should be addressed by the acquirer.

We readily acknowledge that it is impossible to eliminate all areas of risk or even ensure all risk categories are low—we have faced the same issue in our own start-ups and ventures. This process will highlight and at least be able to help your team speak clearly to why a given category stipulates higher risk and why that is acceptable in lieu of mitigation of other risk areas in your project. Not every academic institution will have access to every potential content expert needed for projects like this, but they should have a basic understanding of their capabilities and have a good strategy for identifying how far they are willing to take a project prior to looking for some hand-off or exit opportunity. From there, they can better match required resources to those already “in-house” or those that must be brought in.

The author JS, has been an advisor to many startups and an MIT Venture Mentoring Service Mentor (“VMS”) since 2001. This model for mentorship is the gold standard for mentorship and has proven to be an extremely successful model of mentorship emulated the world over. “Since its founding in 2000, the MIT Venture Mentoring Service has made quite an impact on campus. Employing a unique and rigorous mentoring model, VMS has aided fledgling entrepreneurs in launching dozens of successful companies. However, for the past decade, VMS has also helped launch more than 50 other mentoring programs worldwide, through the VMS Outreach Training Program. See more at “A Model for Mentoring” [8]. Another successful example for a multidisciplinary approach to translational project development and commercialization is the Center for Clinical and Translational Science [9]. They have established internal and external advisory and guidance committees, as well as leadership and administration that has aligned resources and interests of investigators, institutions, and industry. Additional resources complementing your team can be found in programs like NIH and NSF I-Corps and Clinical and Translational Science Awards (CTSA), Commercialization Accelerator Program (CAP), etc. All resources that assist the translational research team’s ability to objectively progress programs to the clinic or effectively de-risk key elements on the critical path for the transition from T0–T1 or T1–T2, etc., should be evaluated and used whenever appropriate.

At the end of this exercise, you should summarize the elements of risk using the questions as a guide and then determine if the risks uncovered will be seen as significant by potential corporate sponsors. This is critical to ensuring your project and rationale are defensible in regards to funding, timing, and development requirements, and will aid in communicating the risk/opportunity of your project to potential sponsors in their own language.

## 4. Incorporation of Manufacturing and Regulatory Requirements

Although explicit details of the commercial development process for drugs and medical devices are also outside the scope of this article, it is helpful to introduce the concepts here as they play a critical role in the steps that tend to immediately follow any academically-initiated translational research project. It is helpful that your team has a general understanding of these guidelines, not so much to be content experts, but to know enough about them to ensure your project follows basic rules and conventions, and to assist in knowing when to seek the appropriate expertise should these guidelines need to be addressed earlier in the process.

In many geographies across the world, including the United States, medical products are regulated by government authorities in various ways and under various agency authorities. In the US, the Food and Drug Administration (FDA) is the overarching regulatory body. These regulatory agencies have defined rules and regulations that you and others developing and manufacturing medical devices must follow, defining how medical devices are classified, and what is required before the products are sold into the marketplace.

The regulatory and development roadmap for medical products are clearly defined and freely available at the European Medicines Agency, European Commission, and FDA websites in the form of Guidance for Industry [10,11,12,13,14]. For example, in the medical device world, the US regulations focused on medical device development are covered under “Design Controls” (CFR 21 820.30 (c)) [11]. We included a short list in the bibliography of several of the many useful references available to guide your understanding of the standard manufacturing, processes as the more of this that can be incorporated or at least considered early on, the higher your likelihood of translational project success.

Specifically, in regards to medical devices, there are many detailed steps, procedures, and documentation that cover: *Processes and procedures*—Quality System, Design Controls, Design History File; *Needs Assessment*—User Needs; *Well-documented Development*—Design and Development, Design Reviews, Design Inputs, Design Outputs; *Testing*—Design Verification, Design Validation; *Manufacturing*—Design Transfer and *Final authorization for Product Launch*. Describing the myriad details and regulations involved in the process of medical device design, development, manufacturing, and testing is beyond the scope of this article and well described elsewhere. The FDA offers Guidance for industry PDFs online of all the documents outlining the process of drug products, medical devices, and diagnostic product development “Cross-Center Final Guidance Documents” [15].

The key challenge with incorporating these regulatory and commercialization guidelines is that academic activities are often hypothesis driven and unregulated, whereas the process of medical product and device development is heavily regulated and defined. The good news is that the regulations established under governmental authority helps to provide a “roadmap” for your team to follow during the product development process, which can help ensure the evidence you collect, the documentation you generate, and the ultimate technology “package” you produce has a higher likelihood of matching those requirements start-ups and larger strategic companies are mandated to follow. This, in turn, helps facilitate an easier hand-off by eliminating a key area of risk these stakeholders are frequently concerned about when exploring the commercialization of academically-generated innovation. We have found that a reasonable approach to this is to establish a basic method for tracking results, design iterations, patent drawings/claims, etc., so that at least these elements are traceable. This is helpful in that when you are offering your innovation for license or acquisition, engineering and quality system experts reviewing your work are easily able to follow changes you made over the course of your research, how you arrived at those changes, how you tested the changes, and what the results of those changes were in the big picture of the innovation.

It is useful to appreciate that there are significant levels of adherence to procedures and establishment and maintenance of documentation companies must maintain to be compliant with regulations. It is also critical to understand that the regulatory arm and resultant audit trail do, in fact, extend to the earliest available records of new technology development. This often involves the original academic research and thus it bears paying attention to these regulatory constraints when developing your translational project in the T1 phase of translational research and development.

## 5. Conclusions

Translational development is best performed in multidisciplinary teams and most often focus on developing and validating concepts that address sizeable market needs at two of the translational “nodes”: (1) translation from basic science to human studies; and (2) translation of new data into better clinical and health decision-making.

Each institution and translational researcher should develop research plans and targets designed to systematically address risks and address value proposition. However, they need to remain flexible to handle projects that were not initiated as part of an innovation or translational pipeline, i.e., arising from traditional academic research pathways, and are identified at a later stage.

Translational medical product development should follow a structured and methodical approach. The market and medical needs for the proposed solution should be validated and measured against the conceptual model of disease and standards of care. We suggest a process as simple as a series of whiteboard sessions to map out the model and identify the areas or risk. Passing these tests, the multidisciplinary team should plan activities addressing and stepping down the identified risk areas and creating a high-level business case for their innovative concept. This approach will evince the value proposition in order to have a much greater chance of success in translating from T0 to T2, as defined by attracting capital, licensing, co-development and M and A opportunities. Products developed in this way will be much more likely to end up in the health care market place solving user-defined unmet needs, as well as having real benefits for patient care.
